# Visual cortex and cerebellum hyperactivation during negative emotion picture stimuli in migraine patients

**DOI:** 10.1038/srep41919

**Published:** 2017-02-09

**Authors:** Mengxing Wang, Jingjing Su, Jilei Zhang, Ying Zhao, Qian Yao, Qiting Zhang, Hui Zhang, Shuo Wang, Ge-Fei Li, Jian-Ren Liu, Xiaoxia Du

**Affiliations:** 1 Shanghai Key Laboratory of Magnetic Resonance and Department of Physics, School of Physics and Materials Science, East China Normal University, Shanghai 200062, China; 2 Department of Neurology and Jiuyuan Municipal Stroke Center, Shanghai Ninth People’s Hospital, Shanghai Jiao Tong University School of Medicine, Shanghai 200011, China

## Abstract

Migraines are a common and undertreated disease and often have psychiatric comorbidities; however, the abnormal mechanism of emotional processing in migraine patients has not been well clarified. This study sought to investigate the different brain functional activation to neutral, positive and negative emotional stimuli between migraine and healthy subjects. Twenty-six adults with migraines and 26 healthy adults, group-matched for sex and age, participated in this experiment. Although there were no significant differences between two groups during the viewing of positive affective pictures vs. neutral affective pictures, there were different activation patterns during the viewing of negative to neutral affective pictures in the two groups; the control group showed both increased and decreased activation patterns, while the migraine subjects showed only increased activation. Negative affective pictures elicited stronger activation than neutral affective pictures in migraineurs, which included the bilateral cerebellum anterior lobe/culmen, the bilateral lingual gyri, the bilateral precuneus and the left cuneus. Our data indicated that migraine patients were hypersensitive to negative stimuli, which might provide clues to aid in the understanding of the pathophysiology and psychiatric comorbidities of migraines.

Migraines are increasingly becoming an important public health concern; about 1 billion people all over the world suffer from migraines^1^. Clearly, migraines are a prominent health-related driver of tremendous economic losses and disability for the world^2^,^3^,^4^. Therefore, approaches to better understand the pathophysiology of migraines are help in finding better diagnostic and treatment plans for patients. Migraines are a common neurologic disorder, characterized by moderate to severe headaches, with a combination of nausea, vomiting, and hypersensitivities to visual, auditory, olfactory, and somatosensory stimuli^5^,^6^. During attacks, many patients are more sensitivity to visual, auditory and olfactory stimuli, and exposure to sensory stimuli can influence and trigger the onset of migraine attacks^7^. Functional magnetic resonance imaging (fMRI) has been used to study the sensory hypersensitivity of migraine by measuring brain responses to visual, heat, ammonia, and painful cutaneous stimulation. Previous fMRI studies consistently demonstrate that migraines exhibit an atypical brain activation in response to olfactory, painful, and visual stimuli^8^,^9^,^10^,^11^. Previous resting state fMRI studies have found many brain areas and networks with abnormal functional connectivity in migraine, which suggested long-term headache attacks would induce brain dysfunction and functional reorganization in migraine^12^,^13^.

Many patients reported that they felt uncomfortable, for example feeling fatigue, stress, and anxiety, before a migraine attack. Migraine patients were significantly different from the healthy control group in the following three dimensions: the lack of positive affect, negative affect, and somatic arousal^14^. Migraineurs also exhibited increased levels of anxiety^15^. Furthermore, migraineurs reported that they more frequently experienced fear and anguish while dreaming when compared with controls^15^. The migraine disability assessment scale scores were correlated with depression subscale scores and stress subscale scores^16^. The literature suggests that migraines are associated with patients’ mental states, especially their negative emotions. Furthermore, a previous study determined that visual, auditory and gustatory stimuli all induced obvious increases in negative affect state scores, and all three types of stimuli evoked a negative affective state and have no significant effect on physical arousal or on trigeminal thermal sensitivity^17^. Thus, stimulation by uncomfortable bodily sensations alone may possibly evoke a negative affective state. In previous fMRI studies, the stimuli were usually uncomfortable, such as ammonia, heat, visual, and odor stimuli, and they were unpleasant and induced hypersensitivity activation^9^,^10^,^11^. Huang *et al*. demonstrated that the responses to non-stressful patterns were similar among controls and migraineurs but the two groups were different in their responses to stressful patterns^18^. Migraineurs had lower discomfort thresholds and increased sensitivity to sensory stimuli^19^. Generally, negative emotion stimuli are also a type of uncomfortable stimuli, and migraineurs may have a lower response threshold to them; thus, migraineurs may be more sensitive to negative events in daily life.

Clinical and pathophysiological evidence has shown that the cerebellum is associated with pain and migraine^20^,^21^,^22^. Pain is a multidimensional experience, which includes sensory, affective, and cognitive processes. Previous studies have suggested that the cerebellum is involved in processing sensorimotor, affective and cognitive information^23^,^24^,^25^. Furthermore, pain-related and emotion-related activation have also been reported in the cerebellum of healthy subjects^26^. A review focused on the cerebellum involvement in migraine, and proposed that inherited calcium channel malfunction leads to hyperexcitability (the main abnormality in migraine) in the brain and cerebellum^20^. There are several previous studies report that migraineurs exhibited structural abnormalities in the cerebellum^21^,^27^,^28^. These structural changes may be accompanied by functional impairment. If migraineurs have dysfunction in the cerebellum, both affective and pain processing would be affected. However, functional information of affective processing in migraineurs is limited.

Emotion pictures as emotional and visual stimuli can induce visual and affective processing. Previous studies have reported that migraineurs were sensitive to visual stimuli; for example; migraineurs exhibited stronger activation in the visual cortex^8^. Thus, we predicted that migraine patients would have abnormalities in visual processing and information integration while viewing negative affective pictures, combined with abnormal affective processing in the cerebellum. In this study, we investigated the whole brain (especially the cerebellum and visual cortex) functional responses of migraine subjects and healthy controls using positive, neutral and negative emotion picture stimuli via fMRI. We hypothesized that negative emotion stimuli could induce hyperactivation in migraineurs due to the hypersensitivity of their brains to negative stimuli.

## Results

### Within-group Activations

**Figure 1:**
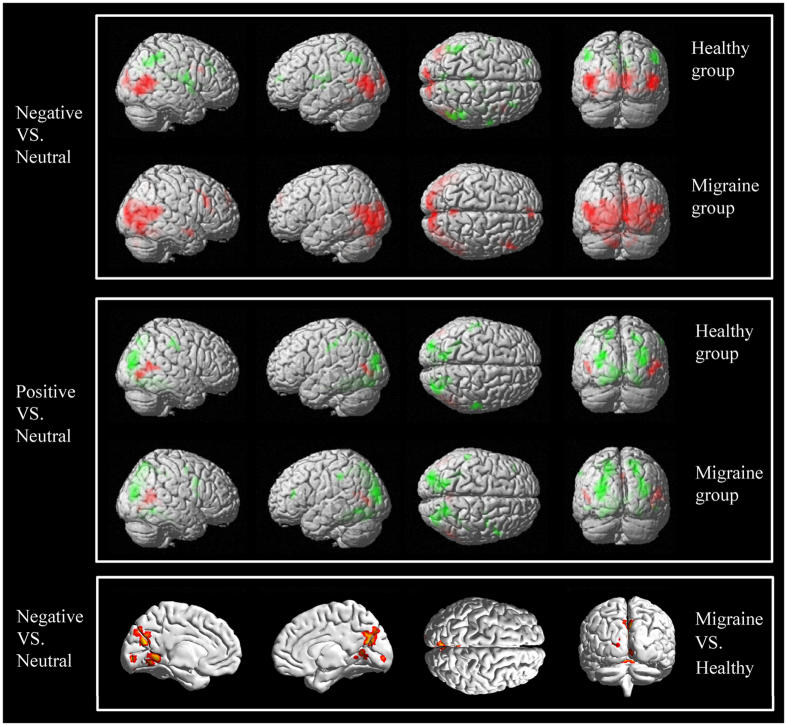
Similar networks of brain regions were activated in the patient group and the control group while viewing positive minus neutral pictures. There were different activation patterns while viewing negative pictures and neutral pictures in the two groups: the control group showed both increased and decreased activation patterns; however, the migraine subjects showed only increased activation. While viewing negative pictures vs. neutral pictures, the patient group showed significantly higher activation than the control group in the bilateral cerebellum anterior lobe/culmen, the bilateral lingual gyri, the bilateral precuneus and the left cuneus. (Red color represents increased activation, and green color represents decreased activation).

Within-group activations for the contrast of negative pictures minus neutral pictures and for the contrast of positive pictures minus neutral pictures are shown in Fig. 1 and Table 1. When comparing positive pictures to neutral pictures, controls had increased activation in the right inferior and superior temporal gyri, the bilateral middle occipital gyrus and the bilateral middle temporal gyrus; furthermore, controls had decreased activation in the bilateral middle occipital gyri, the bilateral cuneus, the bilateral precuneus, the bilateral lingual gyri, the bilateral parahippocampal gyri, the bilateral fusiform gyri, the bilateral cerebellum anterior lobes, the right posterior cingulate cortex, the left superior parietal lobule, and the bilateral postcentral gyri. When comparing the positive pictures to neutral pictures, the migraine group had increased activation in the right superior and middle temporal gyri, the left middle temporal gyrus, and the bilateral precuneus. They also had decreased activation in the bilateral middle occipital gyri, the bilateral cuneus, the bilateral precuneus, the bilateral lingual gyri, the bilateral parahippocampal gyri, the bilateral fusiform gyri, the bilateral cerebellum anterior lobes, the bilateral cerebellum posterior lobes, the left inferior parietal lobule, and the right inferior and middle frontal gyri.

When comparing negative pictures to neutral pictures, the controls showed increased activation in the bilateral middle occipital gyri, the bilateral middle and superior temporal gyri, the bilateral lingual gyri, the bilateral cuneus, the left inferior occipital gyrus, and the right inferior and middle frontal gyri, and they showed decreased activation in the right cingulate gyrus, the right anterior cingulate cortex, the right corpus callosum, the right precuneus and cuneus, the bilateral insular, the bilateral precentral gyri, the left superior and middle frontal gyri, the bilateral medial frontal gyri, the left paracentral lobule, and the bilateral inferior parietal lobules. When comparing negative pictures to neutral pictures, the migraine group had increased activation in the bilateral middle occipital lobes, the bilateral lingual gyri, the bilateral cuneus, the bilateral precuneus, the bilateral fusiform gyri, the left inferior occipital gyrus, the bilateral inferior temporal gyri, the bilateral middle temporal gyri, the bilateral superior temporal gyri, the bilateral parahippocampal gyri, the bilateral posterior cingulate cortex, the bilateral medial and superior frontal gyri, the right inferior and middle frontal gyri, the bilateral cerebellum posterior lobes, the left cerebellum anterior lobe, and the right thalamus; however, the migraine group had no significant decreased activation.

### Between-group Activations

When comparing positive pictures with neutral pictures, there was not apparent difference between the two groups. However, negative pictures elicited stronger activation than neutral pictures in the bilateral cerebellum anterior lobe/culmen, the bilateral lingual gyri, the bilateral precuneus and the left cuneus (see Table 1 and Fig. 1) in migraineurs compared with the controls.

### Clinical and neuropsychological data

Migraine patients showed significantly higher Hamilton Anxiety Scale (HAMA) scores than the healthy controls (P < 0.001), but there was no significant difference between the two groups in Hamilton Depression Scale (HAMD) scores (see Table 2).

## Discussion

In this study, we investigated the different brain activation patterns to positive, neutral and negative emotional stimuli between migraine patients and healthy subjects. When comparing positive pictures to neutral pictures, the control and migraine groups showed similar activation, and the main activation areas were consistent with prior reports^29^,^30^. While viewing the negative pictures vs. neutral pictures, the two groups both showed increased activation in the temporal lobes, the visual cortex and the right prefrontal cortex, which are related to the processing of negative pictures^31^,^32^. However, when viewing negative pictures (minus neutral pictures), the migraine group and control group had different activation patterns: the control group showed both increased and decreased activation, whereas the migraine patients showed only increased activation. The migraine patients exhibited overactivation in response to negative stimuli, whereas the control group suppressed some areas of activation, such as the sensorimotor areas, while viewing negative stimuli compared with neutral stimuli.

Furthermore, compared with the control subjects, the migraineurs exhibited enhanced brain activation in the bilateral cerebellum anterior lobe/culmen, the bilateral lingual gyri, the bilateral precuneus, and the left cuneus while viewing negative pictures compared with neutral pictures. The results showed that the negative stimuli overactivated the cerebellum in migraineurs. The previous studies have suggested that the human cerebellum is connected to cerebral association networks^33^ and that the cerebellum is involved in processing sensorimotor, cognitive and affective information^23^,^24^,^25^. Cerebellar lesions can lead to cerebellar cognitive affective syndrome, which includes affective dysregulation and other processes^24^. Furthermore, the clinical and pathophysiological results have revealed that cerebellum is involved in pain and migraines^20^,^21^,^22^. Several previous studies have reported that migraineurs exhibited volume or microstructural abnormalities in the cerebellum^21^,^27^,^28^. A pervious transcranial magnetic stimulation study showed migraineurs had a significant deficit in cerebellar inhibition compared with controls^34^. In our study, the hyperactivation in the cerebellum potentially suggests that migraines cause decreased inhibition in the cerebellum when patients are faced with negative affective stimuli. The hyperactivation in the cerebellum in migraine patients may be due to genetic factors. Genome-wide association studies have been used to investigate the common forms of migraine, and have identified over ten genetic loci associated with migraines, including areas involved in gene transcription regulation in the cortex and cerebellum^35^. Previous proposals have suggested that genetically driven ion channel dysfunction can result in hyperexcitability in the cerebellum, possibly facilitating the spread of depression waves in the cerebellum^20^. Moulton *et al*. found that unpleasant pictures and heat pain had overlapping cerebellar activation in healthy subjects and suggested that the cerebellum may contain specific regions involved in encoding of generalized aversive processing^26^. Negative and painful stimuli both are aversive stimuli, and they both can active cerebellar responses. The disinhibition of aversive processing in the cerebellum could trigger or aggravate migraines in migraine patients. In our study, the migraineurs showed hyperactivation in the cerebellum while viewing negative stimuli, potentially suggesting that there was less inhibition in the cerebellum of migraineurs, which could be related to the pathophysiological aspects of migraines.

The bilateral lingual gyri, the precuneus and the left cuneus were also overactivated while viewing negative vs. neutral pictures in the migraine group compared with the control group. Our results were consistent with those of a previous review that reported that visual stimuli also induced greater activation in the visual cortex and precuneus in migraineurs than in healthy controls^8^,^9^,^18^,^36^,^37^. Previous studies also demonstrated that migraineurs were significantly more sensitive to light and that the light discomfort threshold was lower in the migraineurs^38^,^39^, and defective neural inhibition in the visual systems of those with migraines has also been reported^40^. In our study, emotion pictures were used as both visual stimuli and affective stimuli. The migraineurs demonstrated no significant differences when viewing positive pictures (minus neutral pictures), but they showed higher activation when viewing negative pictures (minus neutral pictures) than controls. The positive, neutral and negative pictures were similar with regard to the visual features (complexity, color, resolution, size and etc.) but differed in emotional content. Thus, it likely was not the visual stimuli but rather the negative nature of the stimuli that induced the overactivation in the migraine patients, which indicated that the migraineurs could be over-sensitive to negative affective stimuli and have an abnormality in negative emotion processing; this could be one of the reasons for the high rate of psychiatric commodities in migraine patients. It was demonstrated that controls and migraineurs showed similar responses to non-stressful patterns but different responses to stressful patterns^18^. Migraineurs had lower discomfort thresholds and increased sensitivity to sensory stimuli^19^. Given that the negative emotion picture stimuli were also discomforting stimuli, the migraineurs could have a lower response threshold. The negative pictures likely induced overactivation in the visual cortex first and then affected the brain regions associated with emotion processing.

In addition, we found significant increased anxiety (P < 0.001) and slightly increased depression scores (P < 0.1) in the migraine group (see Table 2), although depression and anxiety disorders had been excluded in our study. Migraines are associated with a wide range of medical and psychiatric comorbidities^41^; for example, migraines increase anxiety levels^15^, and migraine disability has been correlated with depression scores and stress scores^16^. Additionally, a personality profile characterized by moderate levels of negative emotion and irritability, together with failures in inhibitory self-regulation, could be induced an increased risk of strict and probable migraines^42^. The literature has suggested that migraines are associated with negative emotion. The discomfort thresholds in response to special sensory stimuli, mechanical stimuli, thermal noxious stimuli and negative emotion stimuli were decreased in migraineurs^6^,^8^. In fact, migraines can cause negative emotions, and negative events can aggravate migraines. Likewise, in daily life, negative events such as fatigue, stress, bad moods, and induced abnormalities in sensory processing and integration might be triggers for migraine attacks, or they could aggravate headaches^6^. Thus, negative emotion may contribute to migraine morbidity and pathology, which can provide some reference for clinical treatment. For example, migraine patients could seek to avoid negative events or effective stimulation in daily life. Additionally, migraine patients may make certain adjustments when faced with negative stimulus events, or they should seek psychological counseling if they cannot avoid negative events. Clinicians should also pay attention to the patient’s psychiatric comorbidities, such as mood, depression and anxiety disorders.

While our research revealed that migraine patients are hypersensitive to negative stimuli, our study had several limitations. Although we used self-reporting questionnaires and interviews to exclude psychiatric disorders, significantly increased anxiety and slightly increased depression scores were found in the migraine group, which could affect activation by negative stimuli. In addition, a larger sample size is needed to investigate the impact of migraine subtypes in brain activation after affective stimulation in migraine patients. Furthermore, the abnormal activation of cerebellum was observed in migraine patients in our study, and the resolution of fMRI should be improved considering the thickness of cerebellum. In future research, high resolution structural and resting-state functional MRI need be used to investigate cerebellum organization and function connectivity in migraineurs, and to further confirm the role of the cerebellum in migraine.

## Conclusion

The present study showed that similar networks of brain regions were activated while viewing positive minus neutral affective pictures but not while viewing negative minus neutral affective pictures in the two groups. The migraine subjects showed hyperactivation in the cerebellum, the lingual gyrus, the precuneus and the cuneus while viewing negative minus neutral affective pictures compared with control subjects. The negative stimuli induced hyperactivation in migraine patients. The results of this study suggest that migraine patients’ hypersensitivities to negative affective stimuli and the dysfunction of the cerebellum and visual cortex may play important roles in the pathophysiology of migraines.

## Materials and Methods

### Subjects

Twenty-six migraine patients (10 males, 16 females) and 26 healthy controls (10 males, 16 females) between the ages of 25 and 59 participated in this study. The patients had a mean age of 40.5 (SD = 9.5) years, and they were recruited from the outpatient clinic of Shanghai Ninth People’s Hospital and diagnosed with migraines based on the International Classification of Headache Disorders (ICHD-II-2004). Of the 26 migraineurs, 20 were classified with migraines without aura, 3 with migraines with aura and 3 with chronic migraines. The control volunteers had a mean age of 41.3 (SD = 10.2) years. All the controls had not suffered from any headaches in the past year, and their family members did not experienced migraines or other headaches. Both patients and controls were right-handed, and all psychiatric and neurological diseases were excluded based on both clinical examinations and MRI structured interviews. The 14-Hamiltion Anxiety Scale (14-HAMA) and 24-Hamilton Depression Scale (24-HAMD) were used to assess the participants’ anxiety depression and depression states. In the migraine group, the disease mean duration was 10.6 (SD = 4.5) years, the mean attack-duration was 13.7 (SD = 11.4) hours, and mean attack-frequency was 3.9 (SD = 1.9) times per month. For further details concerning the patients, please see Table 1.

Both the Ethics Committee of Shanghai Ninth People’s Hospital and the East China Normal University Committee on Human Research (No. HR201603022) approved the study. Each participant signed an informed consent form approved by the committee. All methods were carried out in accordance with the principles outlined in the Declaration of Helsinki, including any relevant details.

### fMRI Paradigm: Affective Picture Task

One-hundred and eight pictures were presented to evoke different emotions from participants. The task consisted of 36 positive, 36 neutral and 36 negative pictures, which were selected from the Chinese affective picture system^43^. Three groups of pictures differed significantly in each valence dimension [F (2,105) = 3048.18, p < 0.001; M ± SD: positive: 7.03 ± 0.17; neutral: 5.01 ± 0.16; negative: 2.45 ± 0.36] and arousal dimension [F (2,105) = 93.00, p < 0.001; M ± SD: positive: 6.08 ± 0.40; neutral: 4.53 ± 0.60; negative: 5.23 ± 0.42]. The experimental paradigm included 3 conditions (positive, neutral and negative). Each condition contained 6 blocks, each block consisted of 6 pictures, and each picture was displayed for 4 s. The blocks were presented pseudo-randomly and were separated by a 10-s fixation crosshair.

All stimuli were presented using a SAMRTEC SA-9900 system (Shenzhen Sinorad Medical Electronics Inc., Shenzhen city, China). The SA-9900 system was to realize synchronization between stimuli presentation and the MRI scanner.

### fMRI Image Acquisition

The MRI data were collected at the Shanghai Key Laboratory of Magnetic Resonance (East China Normal University, Shanghai, China) using a Siemens Trio Tim 3.0 Tesla MRI system. Custom-fit foam pads were placed around the participants’ heads to minimize motion, and a 12-channel head coil was used. The whole-brain anatomical volumes were obtained using a high-resolution T_1_-weighted 3-dimensional magnetization-prepared rapid-acquisition gradient-echo pulse sequence (repetition time (TR) = 2350 ms, echo time (TE) = 2.34 ms, inversion time = 1100 ms, field of view (FOV) = 256 × 256 mm^2^, acquisition matrix = 256 × 256, slice thickness = 1 mm, 50% gap, 192 slices). A T2*-weighted gradient-echo echo-planar-imaging sequence, which was sensitive to blood oxygen level-dependent (BOLD) contrast, were used to collected functional images, with parameters: TR = 2000 ms, TE = 30 ms, FOV = 220 × 220 mm^2^, matrix = 64 × 64, slice thickness = 3.5 mm, 25% gap, 33 slices. There was only one run, and lasted 10 min 28 s, including 6 s dummy scan and 311 volumes.

### fMRI data Analysis

Functional images analysis was performed using statistical parametric mapping software (SPM12; http://www.fil.ion.ucl.ac.uk/spm/software/spm12). The first ten volumes were discarded to make the signal achieve steady-state equilibrium. The preprocessing steps included slice-timing correction, realignment of functional data to each participant’s first image, and co-registration of functional to structural images. All subjects with head movement greater than 3 mm or degrees were excluded from further analysis. The sessions were spatially normalized to the Montreal Neurological Institute (MNI) space. Then Gaussian spatial smoothing (6 mm FWHM) was conducted on the functional images.

In the first-level analysis, a block statistical model was constructed using the general linear model (GLM) with SPM12, and three conditions were modeled for each subject. The contrasts between the positive and neutral conditions and between the negative and neutral conditions were calculated to evaluate the degree of brain activation specific for positive and negative emotional processing, respectively.

The resulting contrast images were used in the second-level group analysis. One-sample and two-sample *t* tests were used to determine within- and between-group differences, respectively. Clusters of activation for display were defined as those surpassing a height threshold of P < 0.001 and significant at the cluster level (P < 0.05, FWE corrected).

In addition, the *t* tests for two independent samples were used to compare the neuropsychological data (HAMD and HAMA scores) between migraine and healthy group using the Statistical Package for the Social Sciences 23.0 (SPSS Inc., Chicago, IL, USA) software. The significance threshold was set at P < 0.05.

## Additional Information

**How to cite this article:** Wang, M. *et al*. Visual cortex and cerebellum hyperactivation during negative emotion picture stimuli in migraine patients. *Sci. Rep.* **7**, 41919; doi: 10.1038/srep41919 (2017).

**Publisher's note:** Springer Nature remains neutral with regard to jurisdictional claims in published maps and institutional affiliations.

## Figures and Tables

**Table 1:** Significant within-group activation while viewing negative compared with neutral pictures in patients with migraines and in control subjects, and the between-group differences in activation.

Cluster	Brain regions	Cluster size	X	Y	Z	T value
**Control group**						
1	Bilateral middle occipital gyri	282 (1644)	−45	−79	2	9.60
	Bilateral middle temporal gyri	426 (1644)	54	−61	5	7.66
	Bilateral superior temporal gyri	182 (1644)	45	−58	17	7.00
	Bilateral lingual gyri	236 (1644)	12	−70	−1	6.07
	Bilateral cuneus	322 (1644)	−12	−94	2	5.86
	Left inferior occipital gyrus	42 (1644)	−39	−76	−10	5.42
2	Right inferior and middle frontal gyri	65	39	17	29	6.13
3	Right cingulate gyrus	70 (331)	3	−25	29	−5.31
	Right corpus callosum	59 (331)	0	−28	20	−4.63
4	Right precuneus and cuneus	226	15	−61	38	−4.89
5	Right insula	184 (352)	42	−16	20	−6.49
	Right precentral gyrus	78 (352)	63	−4	20	−5.21
6	Right insula	184 (352)	42	−16	20	−6.49
	Right precentral gyrus	78 (352)	63	−4	20	−5.21
7	Left insula	108 (213)	−39	−19	17	−6.71
	Left precentral gyrus	28 (213)	−60	−1	20	−4.52
8	Left superior and middle frontal gyrus	46	−27	50	14	−4.61
9	Right anterior cingulate and medial frontal	72	15	29	23	−4.57
10	Left paracentral lobule and medial frontal	80	−9	−34	68	−4.46
11	Left inferior parietal lobule	145	−39	−55	44	−5.72
12	Right inferior parietal lobule	183	45	−40	41	−4.69
**Patients group**						
1	Bilateral middle occipital gyri	709 (4969)	12	−94	11	12.21
	Bilateral inferior temporal gyri	45 (4969)	48	−73	−4	10.86
	Bilateral fusiform gyri	215 (4969)	42	−46	−19	9.12
	Bilateral lingual gyri	714 (4969)	12	−76	−4	9.16
	Bilateral cuneus	707 (4969)	6	−88	26	7.23
	Bilateral middle temporal gyri	531 (4969)	48	−64	11	7.97
	Bilateral superior temporal gyri	383 (4969)	54	−49	11	7.45
	Bilateral cerebellum posterior lobes	430 (4969)	−18	−82	−34	6.27
	Bilateral parahippocampal gyri	90 (4969)	−27	−55	−7	5.70
	Bilateral precuneus	153 (4969)	6	−58	44	6.35
	Bilateral posterior cingulate cortex	85 (4969)	0	−52	26	5.19
	Left inferior occipital gyrus	125 (4969)	33	−88	−10	4.42
	Left cerebellum anterior lobe	69 (4969)	−6	−64	−10	4.33
2	Bilateral medial and superior frontal gyri	103	0	56	29	6.68
3	Right thalamus and parahippocampal gyrus	100	24	−25	2	5.86
4	Right inferior and middle frontal gyri	130	48	23	26	5.39
5	Right middle temporal gyrus	70	54	−1	−16	5.50
**Patient > Control**						
1	Bilateral cerebellum anterior lobe/culmen	96 (153)	−6	−58	−10	4.60
	Bilateral lingual gyri	21 (153)	−15	−58	−1	3.94
2	Left cuneus	36 (81)	−9	−94	8	4.75
	Bilateral lingual gyri	37 (81)	−6	−85	−1	3.87
3	Bilateral precuneus	62 (92)	3	−67	20	4.09
	Left cuneus	25 (92)	6	−67	32	3.83

X, Y, Z are MNI coordinates; An uncorrected P < 0.001 at the voxel level and significant at the cluster level (P < 0.05, FWE corrected) are reported. For example, in “282 (1644)”, 282 represents the voxel number of the bilateral middle occipital gyri, and 1644 represents the voxel number of the cluster including the bilateral middle occipital gyri.

**Table 2:** Clinical data for the patient and control groups.

	Average pain intensity	MIDAS score	HIT-6 score	HAMA score	HAMD score
**Control group**					
Means ± SD	No data	No data	No data	0.9 ± 0.7	1.7 ± 0.9
**Migraine group**					
Means ± SD	7.5 ± 1.2	12.0 ± 5.2	62.9 ± 4.5	3.2 ± 1.5**	2.3 ± 1.2*

MIDAS = Migraine Disability Assessment Scale; HIT-6 = Headache Impact Test; HAMA = Hamilton Anxiety Scale; HAMD = Hamilton Depression Scale. **P < 0.001 migraine patients compared with controls; *P < 0.1 migraine patients compared with controls.
